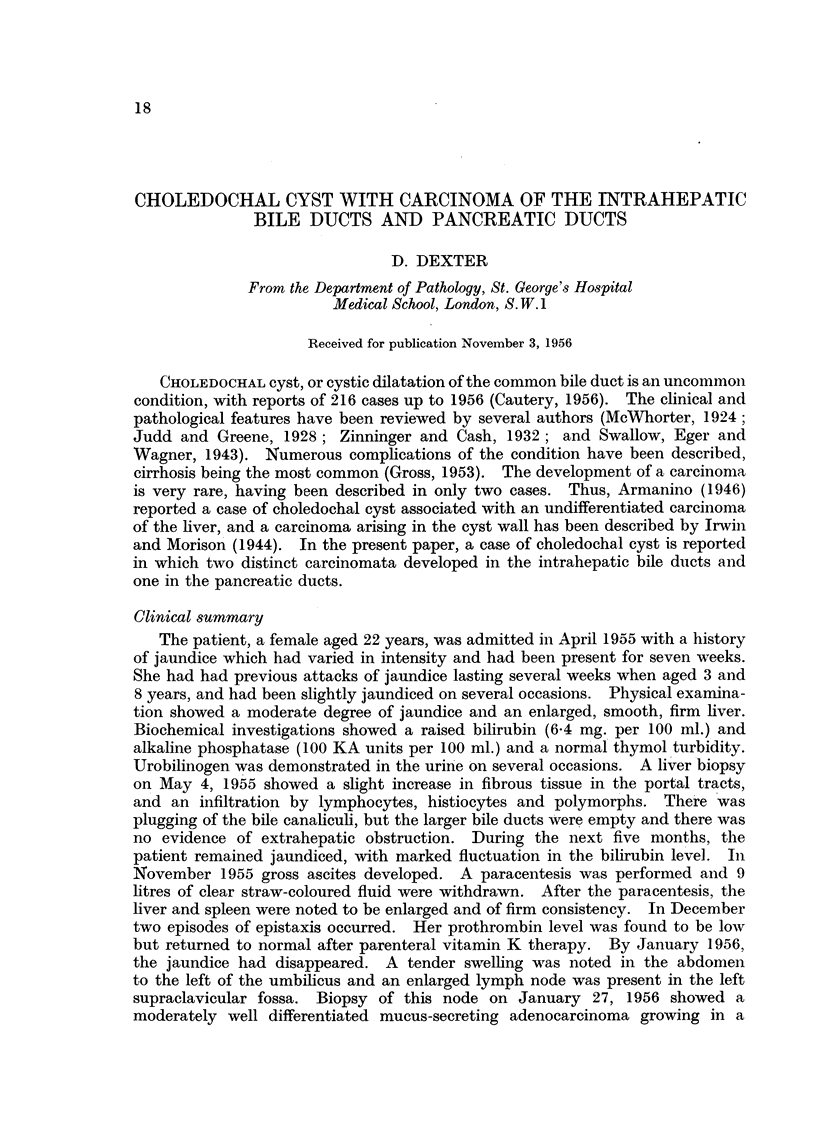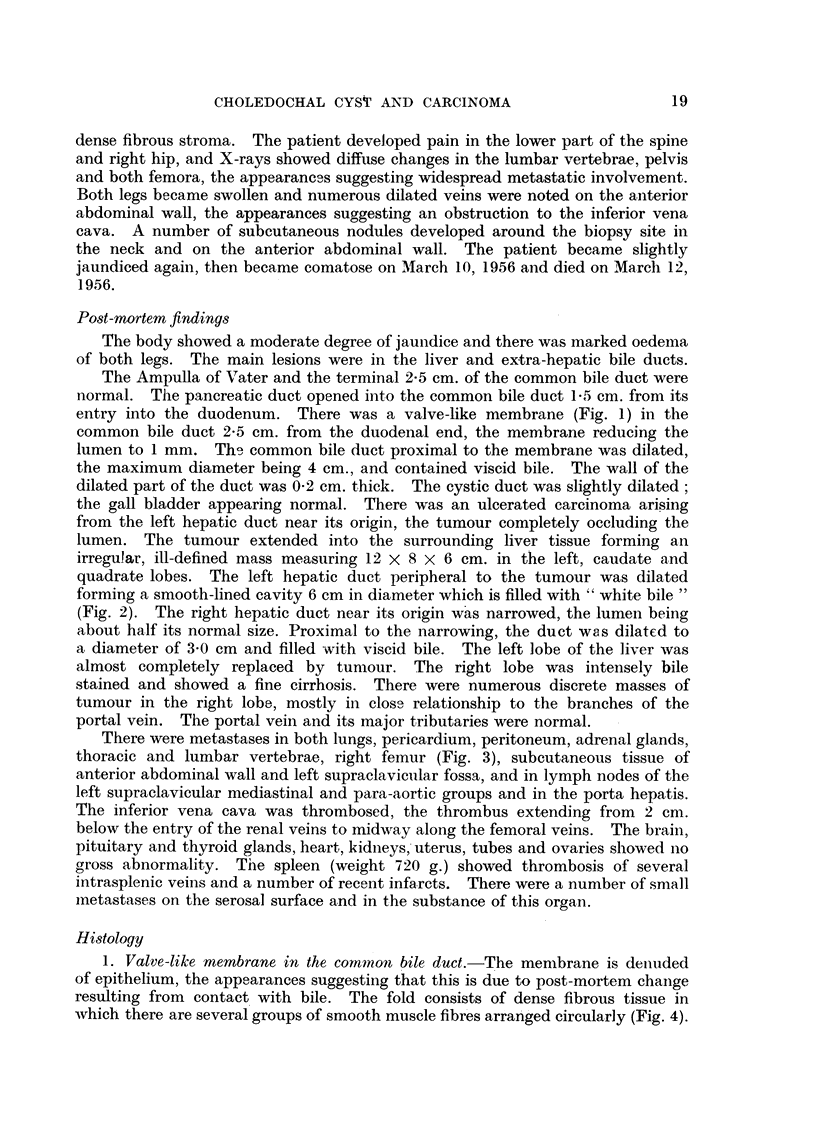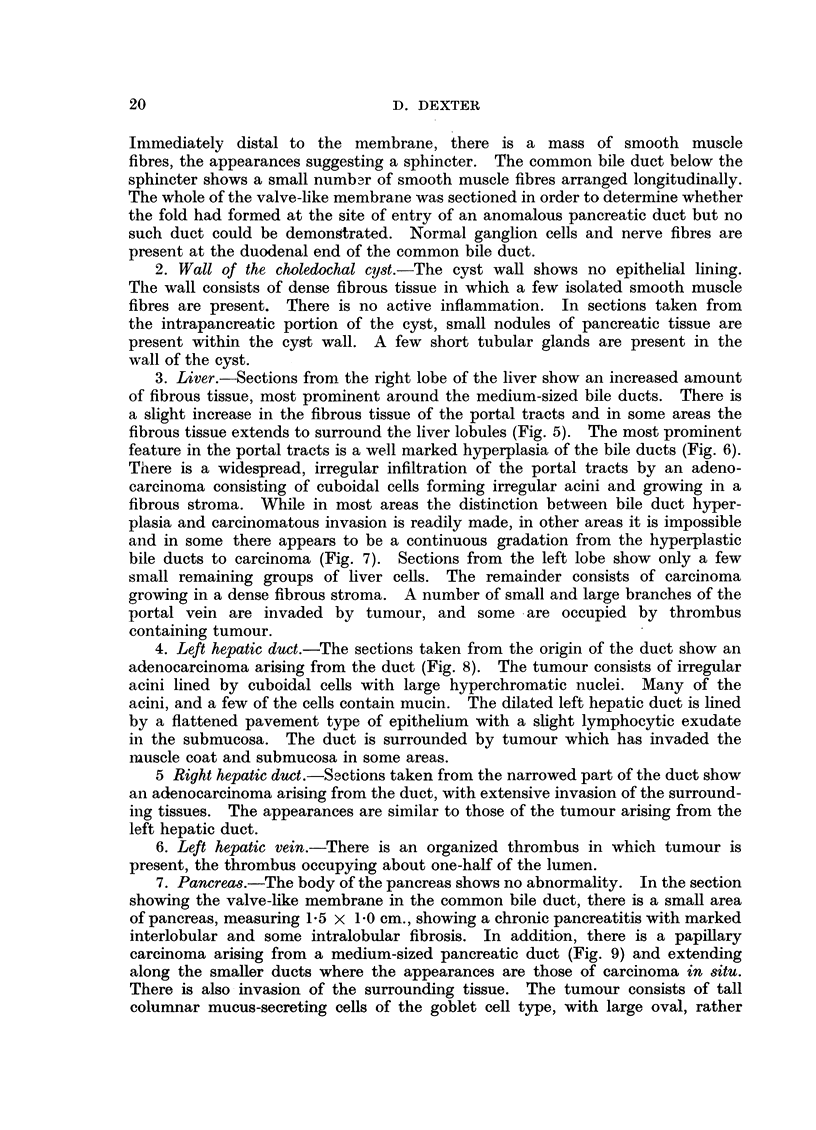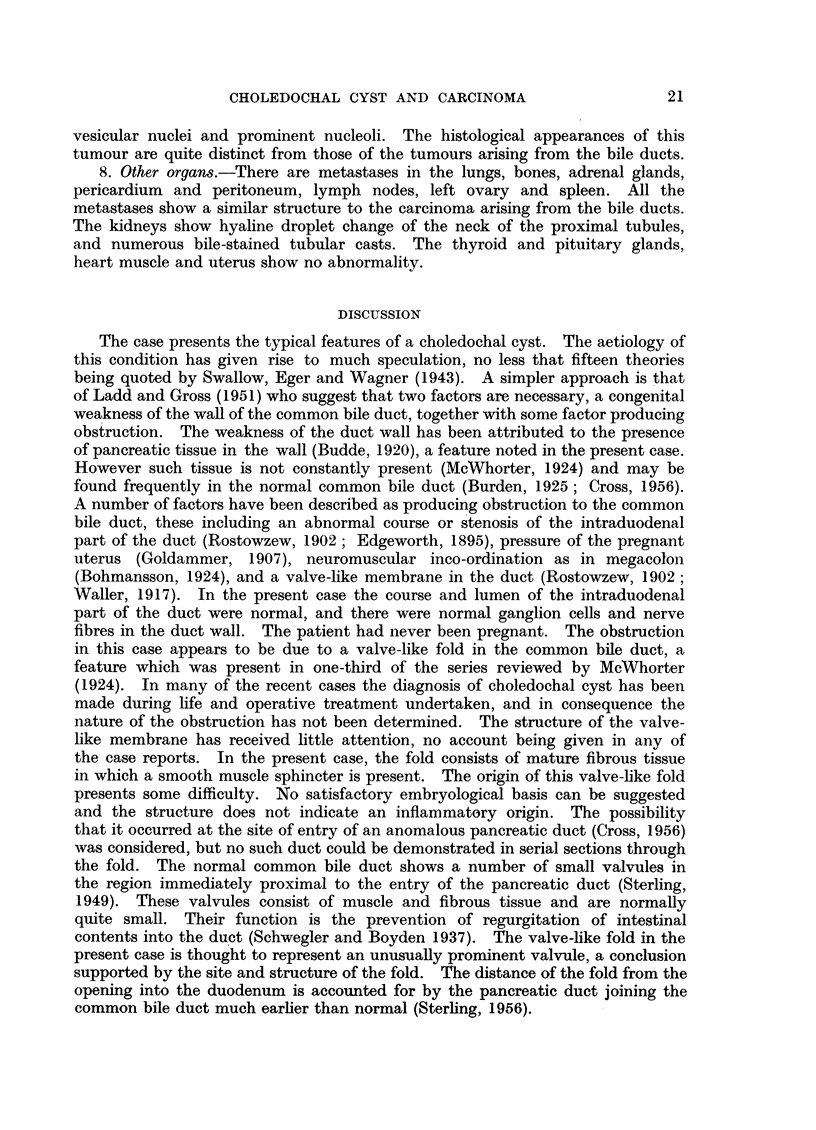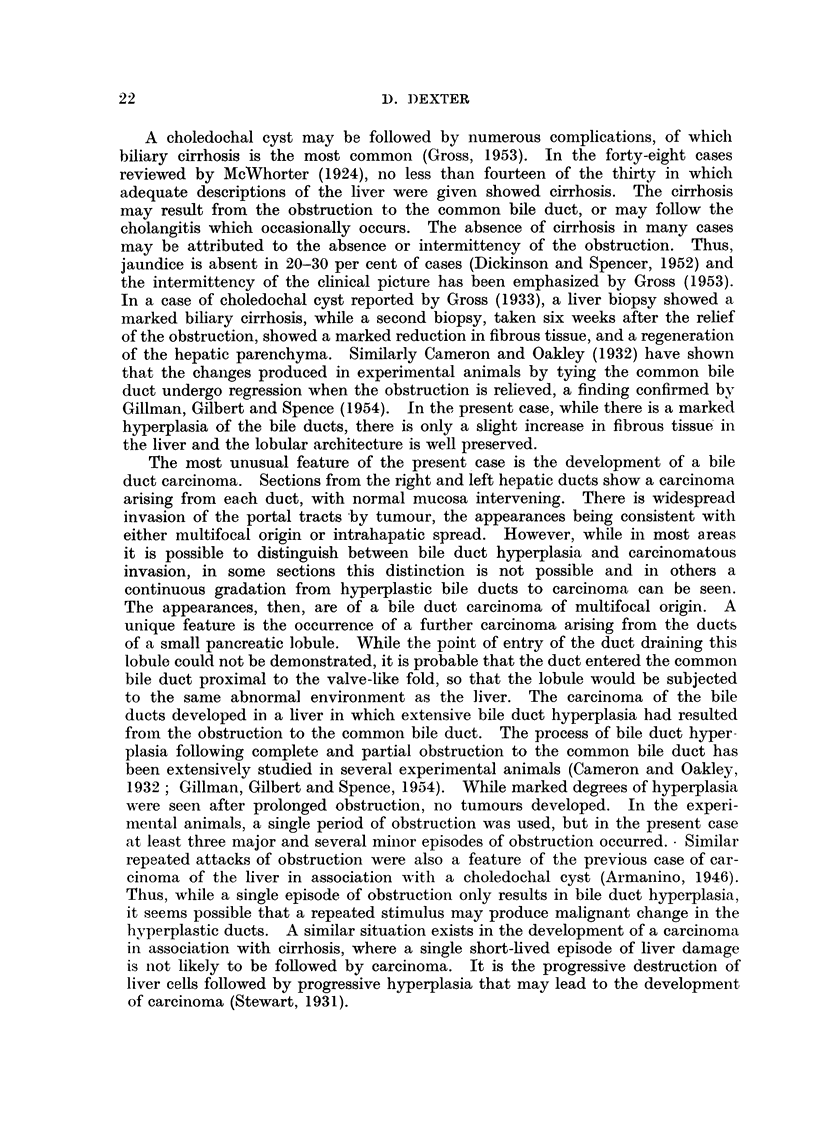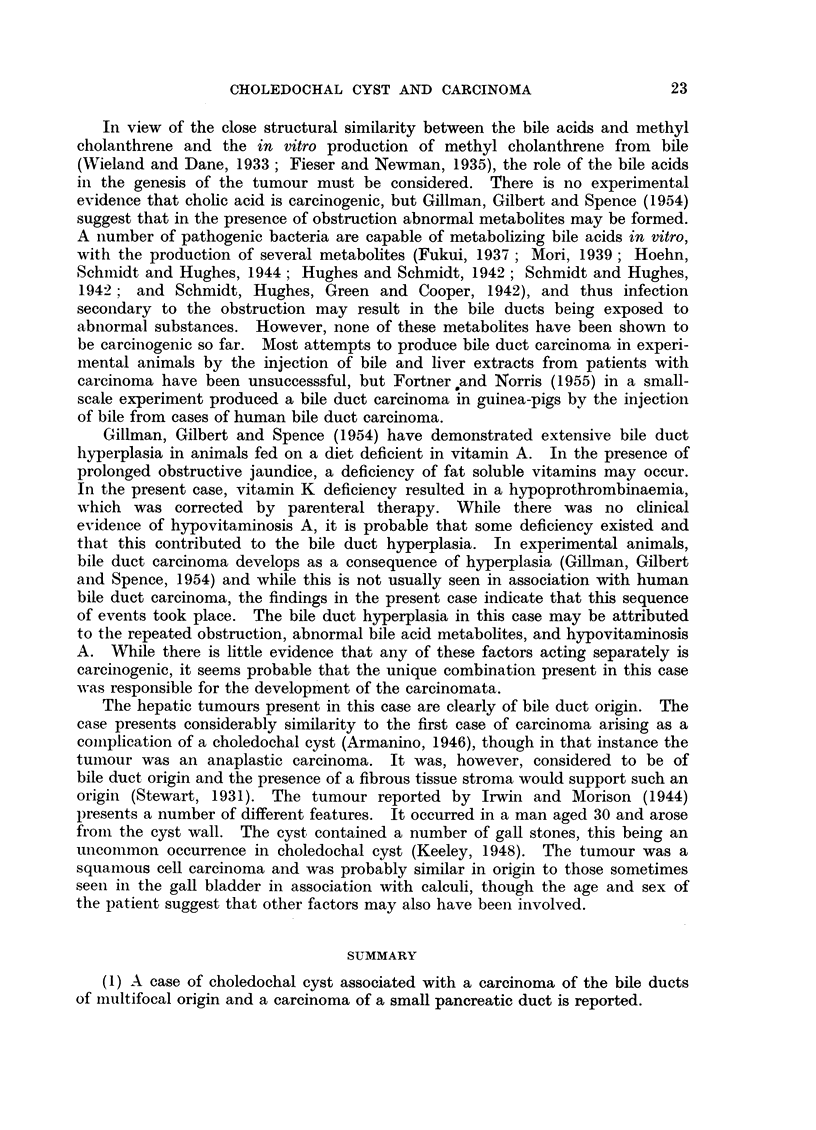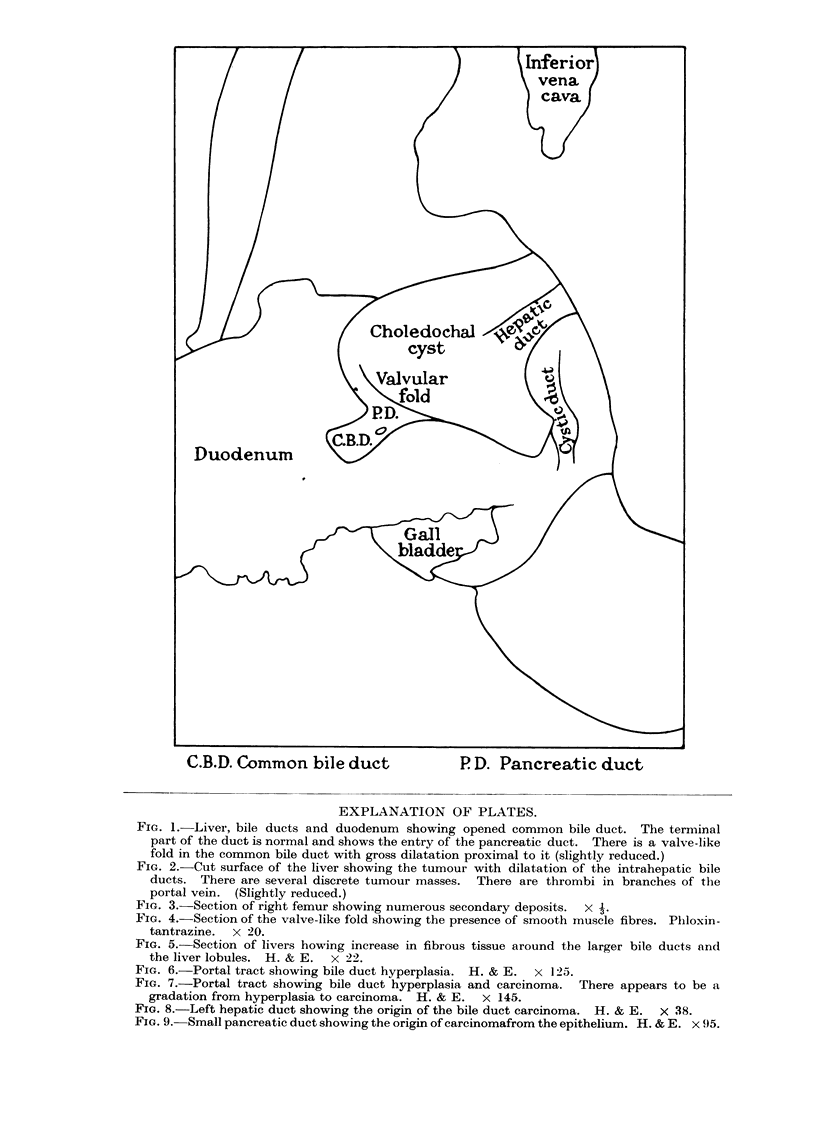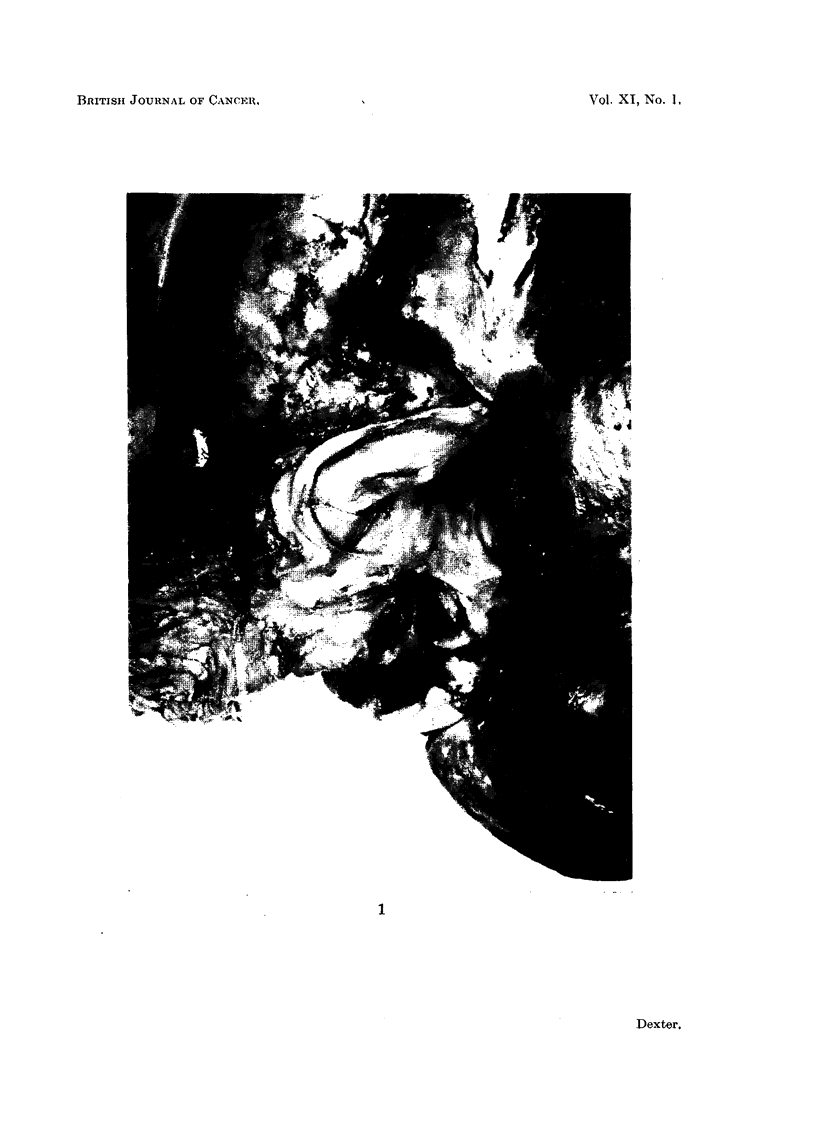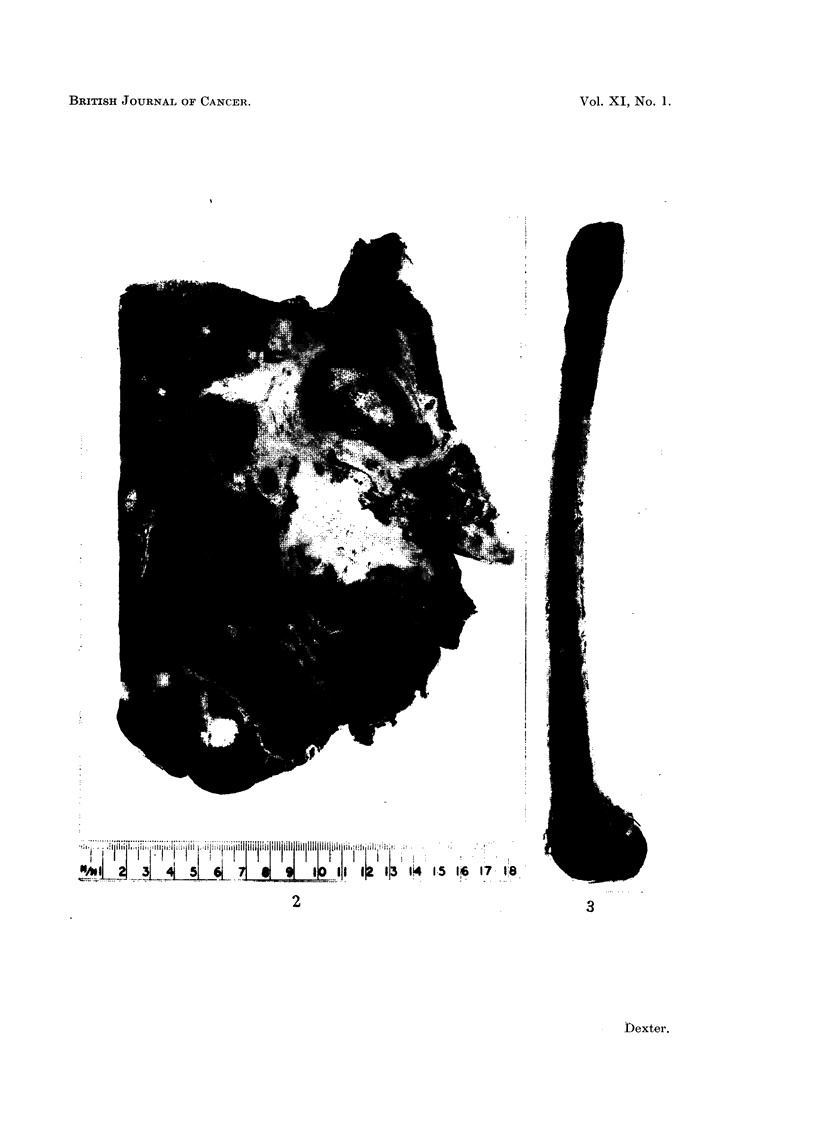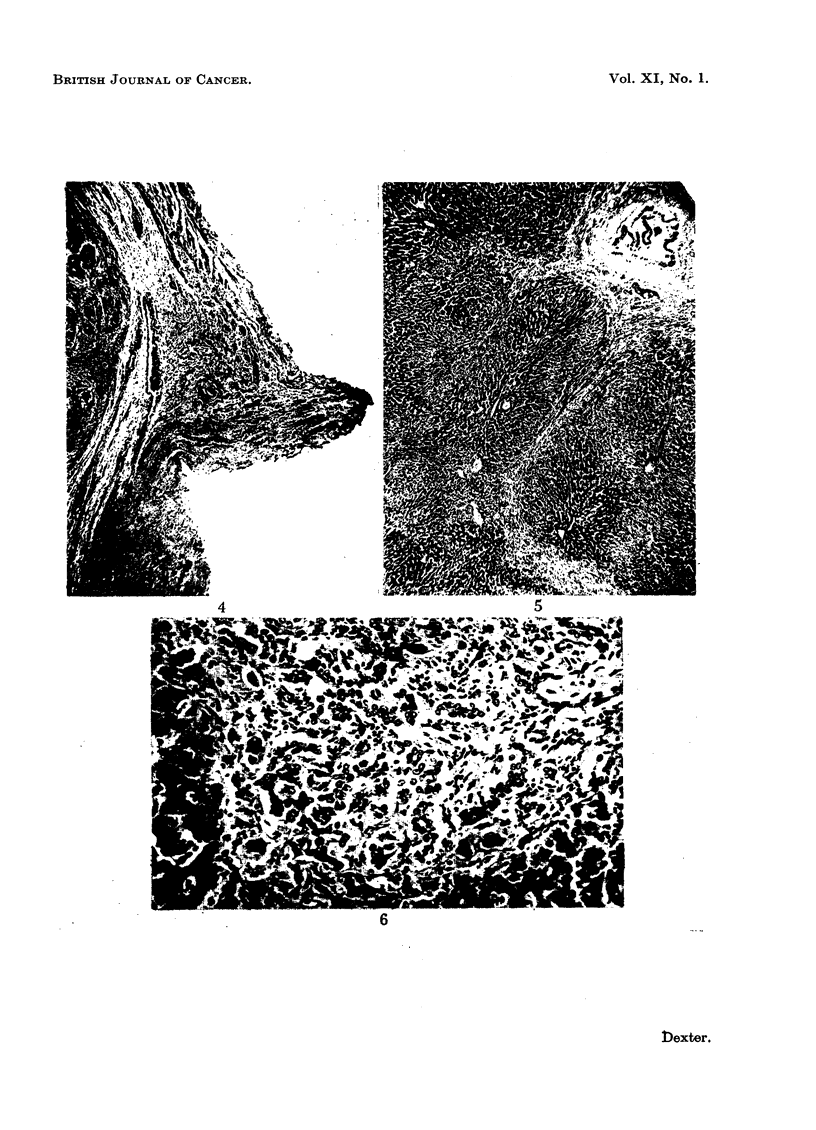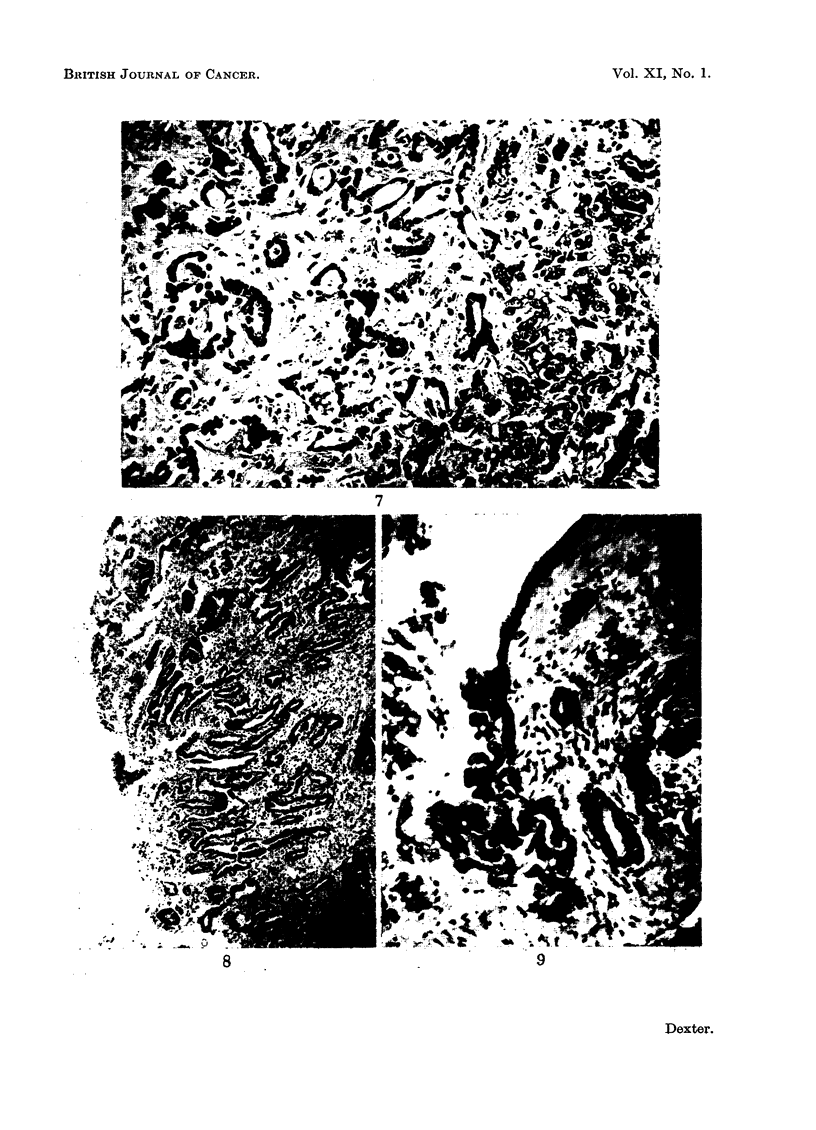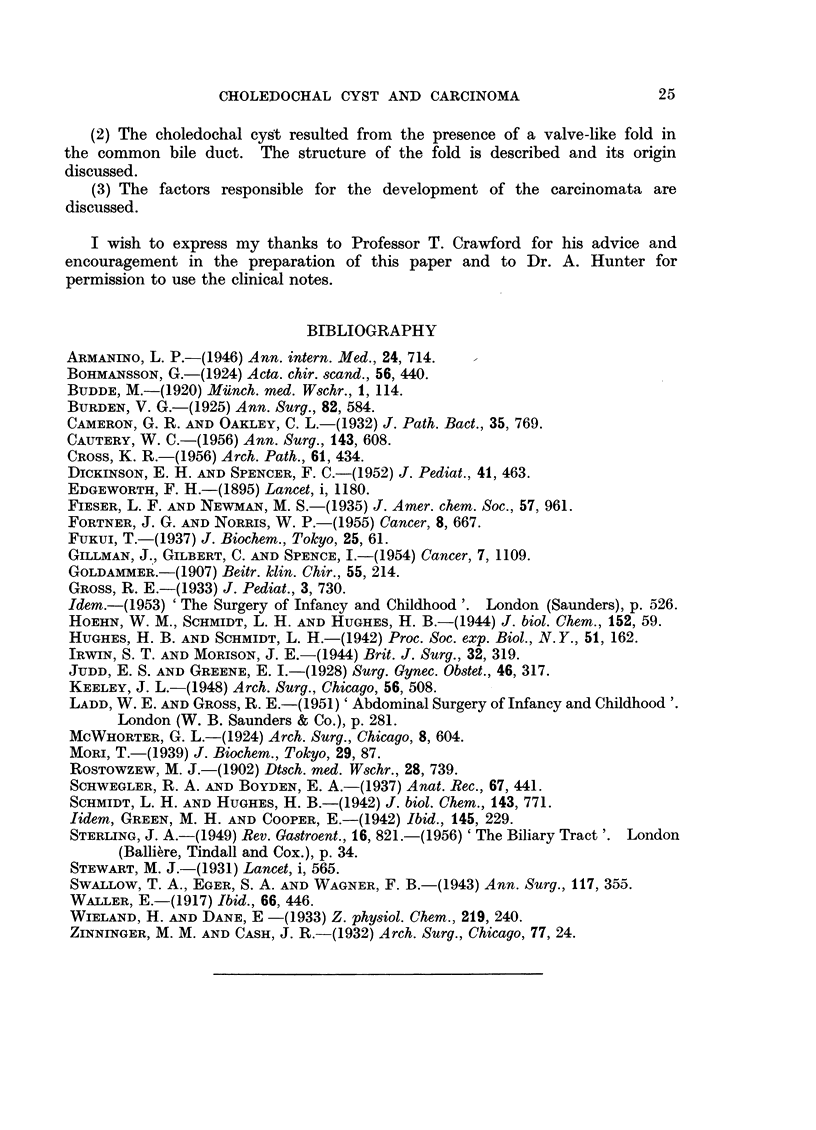# Choledochal Cyst with Carcinoma of the Intrahepatic Bile Ducts and Pancreatic Ducts

**DOI:** 10.1038/bjc.1957.3

**Published:** 1957-03

**Authors:** D. Dexter

## Abstract

**Images:**


### CHOLEDOCHAL CYST WITH CARCINOMA OF THE INTRAHEPATIC BILE DUCTS AND PANCREATIC DUCTS

D. DEXTER

*From the Department of Pathology, St. George's Hospital Medical School, London, S.W.1*

Received for publication November 3, 1956

CHOLEDOCHAL cyst, or cystic dilatation of the common bile duct is an uncommon condition, with reports of 216 cases up to 1956 (Cautery, 1956). The clinical and pathological features have been reviewed by several authors (McWhorter, 1924; Judd and Greene, 1928; Zinninger and Cash, 1932; and Swallow, Eger and Wagner, 1943). Numerous complications of the condition have been described, cirrhosis being the most common (Gross, 1953). The development of a carcinoma is very rare, having been described in only two cases. Thus, Armanino (1946) reported a case of choledochal cyst associated with an undifferentiated carcinoma of the liver, and a carcinoma arising in the cyst wall has been described by Irwin and Morison (1944). In the present paper, a case of choledochal cyst is reported in which two distinct carcinomata developed in the intrahepatic bile ducts and one in the pancreatic ducts.

*Clinical summary*

The patient, a female aged 22 years, was admitted in April 1955 with a history of jaundice which had varied in intensity and had been present for seven weeks. She had had previous attacks of jaundice lasting several weeks when aged 3 and 8 years, and had been slightly jaundiced on several occasions. Physical examination showed a moderate degree of jaundice and an enlarged, smooth, firm liver. Biochemical investigations showed a raised bilirubin (6·4 mg. per 100 ml.) and alkaline phosphatase (100 KA units per 100 ml.) and a normal thymol turbidity. Urobilinogen was demonstrated in the urine on several occasions. A liver biopsy on May 4, 1955 showed a slight increase in fibrous tissue in the portal tracts, and an infiltration by lymphocytes, histiocytes and polymorphs. There was plugging of the bile canaliculi, but the larger bile ducts were empty and there was no evidence of extrahepatic obstruction. During the next five months, the patient remained jaundiced, with marked fluctuation in the bilirubin level. In November 1955 gross ascites developed. A paracentesis was performed and 9 litres of clear straw-coloured fluid were withdrawn. After the paracentesis, the liver and spleen were noted to be enlarged and of firm consistency. In December two episodes of epistaxis occurred. Her prothrombin level was found to be low but returned to normal after parenteral vitamin K therapy. By January 1956, the jaundice had disappeared. A tender swelling was noted in the abdomen to the left of the umbilicus and an enlarged lymph node was present in the left supraclavicular fossa. Biopsy of this node on January 27, 1956 showed a moderately well differentiated mucus-secreting adenocarcinoma growing in a dense fibrous stroma. The patient developed pain in the lower part of the spine and right hip, and X-rays showed diffuse changes in the lumbar vertebrae, pelvis and both femora, the appearances suggesting widespread metastatic involvement. Both legs became swollen and numerous dilated veins were noted on the anterior abdominal wall, the appearances suggesting an obstruction to the inferior vena cava. A number of subcutaneous nodules developed around the biopsy site in the neck and on the anterior abdominal wall. The patient became slightly jaundiced again, then became comatose on March 10, 1956 and died on March 12, 1956.

*Post-mortem findings*

The body showed a moderate degree of jaundice and there was marked oedema of both legs. The main lesions were in the liver and extra-hepatic bile ducts.

The Ampulla of Vater and the terminal 2·5 cm. of the common bile duct were normal. The pancreatic duct opened into the common bile duct 1·5 cm. from its entry into the duodenum. There was a valve-like membrane (Fig. 1) in the common bile duct 2·5 cm. from the duodenal end, the membrane reducing the lumen to 1 mm. The common bile duct proximal to the membrane was dilated, the maximum diameter being 4 cm., and contained viscid bile. The wall of the dilated part of the duct was 0·2 cm. thick. The cystic duct was slightly dilated ; the gall bladder appearing normal. There was an ulcerated carcinoma arising from the left hepatic duct near its origin, the tumour completely occluding the lumen. The tumour extended into the surrounding liver tissue forming an irregular, ill-defined mass measuring 12 × 8 × 6 cm. in the left, caudate and quadrate lobes. The left hepatic duct peripheral to the tumour was dilated forming a smooth-lined cavity 6 cm in diameter which is filled with " white bile " (Fig. 2). The right hepatic duct near its origin was narrowed, the lumen being about half its normal size. Proximal to the narrowing, the duct was dilated to a diameter of 3·0 cm and filled with viscid bile. The left lobe of the liver was almost completely replaced by tumour. The right lobe was intensely bile stained and showed a fine cirrhosis. There were numerous discrete masses of tumour in the right lobe, mostly in close relationship to the branches of the portal vein. The portal vein and its major tributaries were normal.

There were metastases in both lungs, pericardium, peritoneum, adrenal glands, thoracic and lumbar vertebrae, right femur (Fig. 3), subcutaneous tissue of anterior abdominal wall and left supraclavicular fossa, and in lymph nodes of the left supraclavicular mediastinal and para-aortic groups and in the porta hepatis. The inferior vena cava was thrombosed, the thrombus extending from 2 cm. below the entry of the renal veins to midway along the femoral veins. The brain, pituitary and thyroid glands, heart, kidneys, uterus, tubes and ovaries showed no gross abnormality. The spleen (weight 720 g.) showed thrombosis of several intrasplenic veins and a number of recent infarcts. There were a number of small metastases on the serosal surface and in the substance of this organ.

*Histology*

1. *Valve-like membrane in the common bile duct.*—The membrane is denuded of epithelium, the appearances suggesting that this is due to post-mortem change resulting from contact with bile. The fold consists of dense fibrous tissue in which there are several groups of smooth muscle fibres arranged circularly (Fig. 4).

Immediately distal to the membrane, there is a mass of smooth muscle fibres, the appearances suggesting a sphincter. The common bile duct below the sphincter shows a small number of smooth muscle fibres arranged longitudinally. The whole of the valve-like membrane was sectioned in order to determine whether the fold had formed at the site of entry of an anomalous pancreatic duct but no such duct could be demonstrated. Normal ganglion cells and nerve fibres are present at the duodenal end of the common bile duct.

2. *Wall of the choledochal cyst.*—The cyst wall shows no epithelial lining. The wall consists of dense fibrous tissue in which a few isolated smooth muscle fibres are present. There is no active inflammation. In sections taken from the intrapancreatic portion of the cyst, small nodules of pancreatic tissue are present within the cyst wall. A few short tubular glands are present in the wall of the cyst.

3. *Liver.*—Sections from the right lobe of the liver show an increased amount of fibrous tissue, most prominent around the medium-sized bile ducts. There is a slight increase in the fibrous tissue of the portal tracts and in some areas the fibrous tissue extends to surround the liver lobules (Fig. 5). The most prominent feature in the portal tracts is a well marked hyperplasia of the bile ducts (Fig. 6). There is a widespread, irregular infiltration of the portal tracts by an adenocarcinoma consisting of cuboidal cells forming irregular acini and growing in a fibrous stroma. While in most areas the distinction between bile duct hyperplasia and carcinomatous invasion is readily made, in other areas it is impossible and in some there appears to be a continuous gradation from the hyperplastic bile ducts to carcinoma (Fig. 7). Sections from the left lobe show only a few small remaining groups of liver cells. The remainder consists of carcinoma growing in a dense fibrous stroma. A number of small and large branches of the portal vein are invaded by tumour, and some are occupied by thrombus containing tumour.

4. *Left hepatic duct.*—The sections taken from the origin of the duct show an adenocarcinoma arising from the duct (Fig. 8). The tumour consists of irregular acini lined by cuboidal cells with large hyperchromatic nuclei. Many of the acini, and a few of the cells contain mucin. The dilated left hepatic duct is lined by a flattened pavement type of epithelium with a slight lymphocytic exudate in the submucosa. The duct is surrounded by tumour which has invaded the muscle coat and submucosa in some areas.

5 *Right hepatic duct.*—Sections taken from the narrowed part of the duct show an adenocarcinoma arising from the duct, with extensive invasion of the surrounding tissues. The appearances are similar to those of the tumour arising from the left hepatic duct.

6. *Left hepatic vein.*—There is an organized thrombus in which tumour is present, the thrombus occupying about one-half of the lumen.

7. *Pancreas.*—The body of the pancreas shows no abnormality. In the section showing the valve-like membrane in the common bile duct, there is a small area of pancreas, measuring 1·5 × 1·0 cm., showing a chronic pancreatitis with marked interlobular and some intralobular fibrosis. In addition, there is a papillary carcinoma arising from a medium-sized pancreatic duct (Fig. 9) and extending along the smaller ducts where the appearances are those of carcinoma *in situ*. There is also invasion of the surrounding tissue. The tumour consists of tall columnar mucus-secreting cells of the goblet cell type, with large oval, rather vesicular nuclei and prominent nucleoli. The histological appearances of this tumour are quite distinct from those of the tumours arising from the bile ducts.

8. *Other organs.*—There are metastases in the lungs, bones, adrenal glands, pericardium and peritoneum, lymph nodes, left ovary and spleen. All the metastases show a similar structure to the carcinoma arising from the bile ducts. The kidneys show hyaline droplet change of the neck of the proximal tubules, and numerous bile-stained tubular casts. The thyroid and pituitary glands, heart muscle and uterus show no abnormality.

### DISCUSSION

The case presents the typical features of a choledochal cyst. The aetiology of this condition has given rise to much speculation, no less that fifteen theories being quoted by Swallow, Eger and Wagner (1943). A simpler approach is that of Ladd and Gross (1951) who suggest that two factors are necessary, a congenital weakness of the wall of the common bile duct, together with some factor producing obstruction. The weakness of the duct wall has been attributed to the presence of pancreatic tissue in the wall (Budde, 1920), a feature noted in the present case. However such tissue is not constantly present (McWhorter, 1924) and may be found frequently in the normal common bile duct (Burden, 1925 ; Cross, 1956). A number of factors have been described as producing obstruction to the common bile duct, these including an abnormal course or stenosis of the intraduodenal part of the duct (Rostowzew, 1902 ; Edgeworth, 1895), pressure of the pregnant uterus (Goldammer, 1907), neuromuscular inco-ordination as in megacolon (Bohmansson, 1924), and a valve-like membrane in the duct (Rostowzew, 1902 ; Waller, 1917). In the present case the course and lumen of the intraduodenal part of the duct were normal, and there were normal ganglion cells and nerve fibres in the duct wall. The patient had never been pregnant. The obstruction in this case appears to be due to a valve-like fold in the common bile duct, a feature which was present in one-third of the series reviewed by McWhorter (1924). In many of the recent cases the diagnosis of choledochal cyst has been made during life and operative treatment undertaken, and in consequence the nature of the obstruction has not been determined. The structure of the valve-like membrane has received little attention, no account being given in any of the case reports. In the present case, the fold consists of mature fibrous tissue in which a smooth muscle sphincter is present. The origin of this valve-like fold presents some difficulty. No satisfactory embryological basis can be suggested and the structure does not indicate an inflammatory origin. The possibility that it occurred at the site of entry of an anomalous pancreatic duct (Cross, 1956) was considered, but no such duct could be demonstrated in serial sections through the fold. The normal common bile duct shows a number of small valvules in the region immediately proximal to the entry of the pancreatic duct (Sterling, 1949). These valvules consist of muscle and fibrous tissue and are normally quite small. Their function is the prevention of regurgitation of intestinal contents into the duct (Schwegler and Boyden 1937). The valve-like fold in the present case is thought to represent an unusually prominent valvule, a conclusion supported by the site and structure of the fold. The distance of the fold from the opening into the duodenum is accounted for by the pancreatic duct joining the common bile duct much earlier than normal (Sterling, 1956).

A choledochal cyst may be followed by numerous complications, of which biliary cirrhosis is the most common (Gross, 1953). In the forty-eight cases reviewed by McWhorter (1924), no less than fourteen of the thirty in which adequate descriptions of the liver were given showed cirrhosis. The cirrhosis may result from the obstruction to the common bile duct, or may follow the cholangitis which occasionally occurs. The absence of cirrhosis in many cases may be attributed to the absence or intermittency of the obstruction. Thus, jaundice is absent in 20–30 per cent of cases (Dickinson and Spencer, 1952) and the intermittency of the clinical picture has been emphasized by Gross (1953). In a case of choledochal cyst reported by Gross (1933), a liver biopsy showed a marked biliary cirrhosis, while a second biopsy, taken six weeks after the relief of the obstruction, showed a marked reduction in fibrous tissue, and a regeneration of the hepatic parenchyma. Similarly Cameron and Oakley (1932) have shown that the changes produced in experimental animals by tying the common bile duct undergo regression when the obstruction is relieved, a finding confirmed by Gillman, Gilbert and Spence (1954). In the present case, while there is a marked hyperplasia of the bile ducts, there is only a slight increase in fibrous tissue in the liver and the lobular architecture is well preserved.

The most unusual feature of the present case is the development of a bile duct carcinoma. Sections from the right and left hepatic ducts show a carcinoma arising from each duct, with normal mucosa intervening. There is widespread invasion of the portal tracts by tumour, the appearances being consistent with either multifocal origin or intrahapatic spread. However, while in most areas it is possible to distinguish between bile duct hyperplasia and carcinomatous invasion, in some sections this distinction is not possible and in others a continuous gradation from hyperplastic bile ducts to carcinoma can be seen. The appearances, then, are of a bile duct carcinoma of multifocal origin. A unique feature is the occurrence of a further carcinoma arising from the ducts of a small pancreatic lobule. While the point of entry of the duct draining this lobule could not be demonstrated, it is probable that the duct entered the common bile duct proximal to the valve-like fold, so that the lobule would be subjected to the same abnormal environment as the liver. The carcinoma of the bile ducts developed in a liver in which extensive bile duct hyperplasia had resulted from the obstruction to the common bile duct. The process of bile duct hyperplasia following complete and partial obstruction to the common bile duct has been extensively studied in several experimental animals (Cameron and Oakley, 1932 ; Gillman, Gilbert and Spence, 1954). While marked degrees of hyperplasia were seen after prolonged obstruction, no tumours developed. In the experimental animals, a single period of obstruction was used, but in the present case at least three major and several minor episodes of obstruction occurred. Similar repeated attacks of obstruction were also a feature of the previous case of carcinoma of the liver in association with a choledochal cyst (Armanino, 1946). Thus, while a single episode of obstruction only results in bile duct hyperplasia, it seems possible that a repeated stimulus may produce malignant change in the hyperplastic ducts. A similar situation exists in the development of a carcinoma in association with cirrhosis, where a single short-lived episode of liver damage is not likely to be followed by carcinoma. It is the progressive destruction of liver cells followed by progressive hyperplasia that may lead to the development of carcinoma (Stewart, 1931).

In view of the close structural similarity between the bile acids and methyl cholanthrene and the *in vitro* production of methyl cholanthrene from bile (Wieland and Dane, 1933 ; Fieser and Newman, 1935), the role of the bile acids in the genesis of the tumour must be considered. There is no experimental evidence that cholic acid is carcinogenic, but Gillman, Gilbert and Spence (1954) suggest that in the presence of obstruction abnormal metabolites may be formed. A number of pathogenic bacteria are capable of metabolizing bile acids *in vitro*, with the production of several metabolites (Fukui, 1937 ; Mori, 1939 ; Hoehn, Schmidt and Hughes, 1944 ; Hughes and Schmidt, 1942 ; Schmidt and Hughes, 1942 ; and Schmidt, Hughes, Green and Cooper, 1942), and thus infection secondary to the obstruction may result in the bile ducts being exposed to abnormal substances. However, none of these metabolites have been shown to be carcinogenic so far. Most attempts to produce bile duct carcinoma in experimental animals by the injection of bile and liver extracts from patients with carcinoma have been unsuccesssful, but Fortner and Norris (1955) in a small-scale experiment produced a bile duct carcinoma in guinea-pigs by the injection of bile from cases of human bile duct carcinoma.

Gillman, Gilbert and Spence (1954) have demonstrated extensive bile duct hyperplasia in animals fed on a diet deficient in vitamin A. In the presence of prolonged obstructive jaundice, a deficiency of fat soluble vitamins may occur. In the present case, vitamin K deficiency resulted in a hypoprothrombinaemia, which was corrected by parenteral therapy. While there was no clinical evidence of hypovitaminosis A, it is probable that some deficiency existed and that this contributed to the bile duct hyperplasia. In experimental animals, bile duct carcinoma develops as a consequence of hyperplasia (Gillman, Gilbert and Spence, 1954) and while this is not usually seen in association with human bile duct carcinoma, the findings in the present case indicate that this sequence of events took place. The bile duct hyperplasia in this case may be attributed to the repeated obstruction, abnormal bile acid metabolites, and hypovitaminosis A. While there is little evidence that any of these factors acting separately is carcinogenic, it seems probable that the unique combination present in this case was responsible for the development of the carcinomata.

The hepatic tumours present in this case are clearly of bile duct origin. The case presents considerably similarity to the first case of carcinoma arising as a complication of a choledochal cyst (Armanino, 1946), though in that instance the tumour was an anaplastic carcinoma. It was, however, considered to be of bile duct origin and the presence of a fibrous tissue stroma would support such an origin (Stewart, 1931). The tumour reported by Irwin and Morison (1944) presents a number of different features. It occurred in a man aged 30 and arose from the cyst wall. The cyst contained a number of gall stones, this being an uncommon occurrence in choledochal cyst (Keeley, 1948). The tumour was a squamous cell carcinoma and was probably similar in origin to those sometimes seen in the gall bladder in association with calculi, though the age and sex of the patient suggest that other factors may also have been involved.

### SUMMARY

(1) A case of choledochal cyst associated with a carcinoma of the bile ducts of multifocal origin and a carcinoma of a small pancreatic duct is reported.

C.B.D. Common bile duct P. D. Pancreatic duct

---

EXPLANATION OF PLATES.

Fig. 1.—Liver, bile ducts and duodenum showing opened common bile duct. The terminal part of the duct is normal and shows the entry of the pancreatic duct. There is a valve-like fold in the common bile duct with gross dilatation proximal to it (slightly reduced.)

Fig. 2.—Cut surface of the liver showing the tumour with dilatation of the intrahepatic bile ducts. There are several discrete tumour masses. There are thrombi in branches of the portal vein. (Slightly reduced.)

Fig. 3.—Section of right femur showing numerous secondary deposits. $\times \frac{1}{3}$.

Fig. 4.—Section of the valve-like fold showing the presence of smooth muscle fibres. Phloxin-tantrazine. $\times$ 20.

Fig. 5.—Section of livers howing increase in fibrous tissue around the larger bile ducts and the liver lobules. H. & E. $\times$ 22.

Fig. 6.—Portal tract showing bile duct hyperplasia. H. & E. $\times$ 125.

Fig. 7.—Portal tract showing bile duct hyperplasia and carcinoma. There appears to be a gradation from hyperplasia to carcinoma. H. & E. $\times$ 145.

Fig. 8.—Left hepatic duct showing the origin of the bile duct carcinoma. H. & E. $\times$ 38.

Fig. 9.—Small pancreatic duct showing the origin of carcinomafrom the epithelium. H. & E. $\times$ 95.

1

2

3

Dexter.

4

5

6

7

8

9

Dexter.

(2) The choledochal cyst resulted from the presence of a valve-like fold in the common bile duct. The structure of the fold is described and its origin discussed.

(3) The factors responsible for the development of the carcinomata are discussed.

I wish to express my thanks to Professor T. Crawford for his advice and encouragement in the preparation of this paper and to Dr. A. Hunter for permission to use the clinical notes.

### BIBLIOGRAPHY

ARMANINO, L. P.—(1946) *Ann. intern. Med.*, **24**, 714.
BOHMANSSON, G.—(1924) *Acta. chir. scand.*, **56**, 440.
BUDDE, M.—(1920) *Münch. med. Wschr.*, **1**, 114.
BURDEN, V. G.—(1925) *Ann. Surg.*, **82**, 584.
CAMERON, G. R. AND OAKLEY, C. L.—(1932) *J. Path. Bact.*, **35**, 769.
CAUTERY, W. C.—(1956) *Ann. Surg.*, **143**, 608.
CROSS, K. R.—(1956) *Arch. Path.*, **61**, 434.
DICKINSON, E. H. AND SPENCER, F. C.—(1952) *J. Pediat.*, **41**, 463.
EDGEWORTH, F. H.—(1895) *Lancet*, i, 1180.
FIESER, L. F. AND NEWMAN, M. S.—(1935) *J. Amer. chem. Soc.*, **57**, 961.
FORTNER, J. G. AND NORRIS, W. P.—(1955) *Cancer*, **8**, 667.
FUKUI, T.—(1937) *J. Biochem., Tokyo*, **25**, 61.
GILLMAN, J., GILBERT, C. AND SPENCE, I.—(1954) *Cancer*, **7**, 1109.
GOLDAMMER.—(1907) *Beitr. klin. Chir.*, **55**, 214.
GROSS, R. E.—(1933) *J. Pediat.*, **3**, 730.
*Idem.*—(1953) 'The Surgery of Infancy and Childhood'. London (Saunders), p. 526.
HOEHN, W. M., SCHMIDT, L. H. AND HUGHES, H. B.—(1944) *J. biol. Chem.*, **152**, 59.
HUGHES, H. B. AND SCHMIDT, L. H.—(1942) *Proc. Soc. exp. Biol., N.Y.*, **51**, 162.
IRWIN, S. T. AND MORISON, J. E.—(1944) *Brit. J. Surg.*, **32**, 319.
JUDD, E. S. AND GREENE, E. I.—(1928) *Surg. Gynec. Obstet.*, **46**, 317.
KEELEY, J. L.—(1948) *Arch. Surg., Chicago*, **56**, 508.
LADD, W. E. AND GROSS, R. E.—(1951) 'Abdominal Surgery of Infancy and Childhood'. London (W. B. Saunders & Co.), p. 281.
McWHORTER, G. L.—(1924) *Arch. Surg., Chicago*, **8**, 604.
MORI, T.—(1939) *J. Biochem., Tokyo*, **29**, 87.
ROSTOWZEW, M. J.—(1902) *Dtsch. med. Wschr.*, **28**, 739.
SCHWEGLER, R. A. AND BOYDEN, E. A.—(1937) *Anat. Rec.*, **67**, 441.
SCHMIDT, L. H. AND HUGHES, H. B.—(1942) *J. biol. Chem.*, **143**, 771.
*Iidem*, GREEN, M. H. AND COOPER, E.—(1942) *Ibid.*, **145**, 229.
STERLING, J. A.—(1949) *Rev. Gastroent.*, **16**, 821.—(1956) 'The Biliary Tract'. London (Ballière, Tindall and Cox.), p. 34.
STEWART, M. J.—(1931) *Lancet*, i, 565.
SWALLOW, T. A., EGER, S. A. AND WAGNER, F. B.—(1943) *Ann. Surg.*, **117**, 355.
WALLER, E.—(1917) *Ibid.*, **66**, 446.
WIELAND, H. AND DANE, E —(1933) *Z. physiol. Chem.*, **219**, 240.
ZINNINGER, M. M. AND CASH, J. R.—(1932) *Arch. Surg., Chicago*, **77**, 24.